# Effectiveness of THC-containing cannabis for inflammatory bowel disease: a systematic review

**DOI:** 10.1186/s42238-026-00456-2

**Published:** 2026-06-03

**Authors:** Sebastian Jugl, Silvia Perez-Vilar, Steven Galati, Sukhminder K. Sandhu, Hung-Kai Chen, Shao-Hsuan Chang, Yehua Wang, Thorben Kurzbach, Lauren Adkins, Jingchuan Guo, Yan Wang, Almut G. Winterstein, Amie Goodin

**Affiliations:** 1 Department of Pharmaceutical Outcomes and Policy, University of Florida, 6018 Malachowsky Hall, Gainesville, FL 32611 USA; 2 Center for Drug Evaluation & Safety (CoDES), Gainesville, FL USA; 3 Consortium for Medical Marijuana Clinical Outcomes Research, Gainesville, FL USA; 4 Center for Drug Evaluation and Research, US Food and Drug Administration, Silver Spring, MD USA; 5 Health Science Center Libraries, University of Florida, Gainesville, FL USA; 6 Department of Epidemiology, University of Florida, Gainesville, FL USA

**Keywords:** Inflammatory Bowel Disease, Delta-9-tetrahydrocannabinol, Medical Marijuana, Medical Cannabis, Ulcerative Colitis, Crohn’s Disease

## Abstract

**Background:**

Patients with inflammatory bowel disease (IBD) increasingly use cannabis. Studies investigating the effectiveness and safety of cannabis as a therapeutic option for IBD have reported conflicting findings, and recent reviews have not incorporated evidence from real-world studies. This systematic review assessed controlled non-interventional studies and interventional trials evaluating the effectiveness of delta-9-tetrahydrocannabinol (THC)-containing cannabis products in patients with IBD.

**Methods:**

The systematic review procedures—including search strategy across four databases, literature screening, data extraction, qualitative result synthesis, and risk of bias assessment—followed the Cochrane Handbook and adhered to PRISMA-compliant reporting. We used the GRADE approach to rate evidence quality for each outcome. Inclusion criteria: peer-reviewed research on effectiveness or safety of cannabis as treatment modality, control group must be present, quantitative measure of effect/safety on IBD. Exclusion criteria: published before 2000, non-English, non-human research, non-cannabis/THC exposures, < 0.3% THC exposure, topical formulations.

**Results:**

Four randomized clinical trials (RCTs), and four non-interventional studies met all inclusion criteria. Non-interventional studies exhibited serious or critical risk of bias, leading to their exclusion from evidence ratings. The four RCTs demonstrated high risk of bias, although their evidence quality ratings varied. RCTs assessed adverse events; however, limited safety information was provided. Improvements in one trial were reported for bloating (*n* = 56) and appetite (*n* = 56), both rated as low evidence quality. Results for clinical disease activity scores (4 trials, *n* = 180), daily function, general well-being, general effect on health (1 trial, *n* = 56), bowel movement frequency (3 trials, *n* = 148), generic quality of life (3 trials, *n* = 122) and pain (2 trials, *n* = 88) were mixed. The outcomes of remission (3 trials, *n* = 150), weight (1 trial, *n* = 56), IBDQ (1 trial, *n* = 60), endoscopic scores (2 trials, *n* = 88), and nausea (1 trial, *n* = 56), consistently failed to differ meaningfully between intervention and control. The evidence quality rating for these null or inconclusive results varied from low to medium, with the exception of weight, rated as high, though no effect was reported.

**Conclusions:**

Current evidence is inconclusive as to whether THC-containing cannabis products might have a positive effect in IBD, given heterogenous findings and mostly low-to-moderate evidence quality. Higher quality cannabis research is needed given widespread utilization.

**Trial registration:**

PROSPERO: CRD42023411910.

**Supplementary Information:**

The online version contains supplementary material available at 10.1186/s42238-026-00456-2.

## Background

Management of inflammatory bowel disease (IBD), including its most common forms—Crohn’s disease and ulcerative colitis—typically involves a combination of medication, lifestyle modifications, and surgery. (Rubin et al. 2019; Lichtenstein et al. 2018) The goal of a clinical remission is only achieved for a subset of patients over the lifetime disease course, (Rubin et al. 2019; Lichtenstein et al. 2018; Danese et al. 2015) emphasizing the need for new treatment modalities. Recent surveys suggest that 12–38% percent of patients with IBD use cannabis in countries with legal access, with more than half reporting use to relieve their IBD-related symptoms. (Lal et al. 2011; Velez-Santiago et al. 2023) Pre-clinical research has demonstrated that certain cannabinoids (including those contained in the cannabis plant) activate cannabinoid receptor (CBR) 1, which decreases release of acetylcholine, reducing gut motility, and downregulates transient receptor potential vanilloid 1 (TRPV1), potentially alleviating visceral pain. (Pesce et al. 2018; Landi et al. 2002; Esfandyari et al. 2007; Hong et al. 2009) CBR 2 agonists, including cannabidiol and fatty acid amide hydrolase (FAAH) reduce colonic inflammation in animal studies, further suggesting therapeutic potential of cannabis for IBD. (Couch et al. 2018).

Several systematic reviews have evaluated the role of cannabis and cannabinoids in the treatment of patients with IBD and reported mixed findings. Two Cochrane systematic reviews of randomized trials in patients with ulcerative colitis and Crohn’s disease concluded that effectiveness remains uncertain. (Kafil et al. 2018a; 2018b) A subsequent systematic review including both trials and non-randomized studies reported no evidence of improvements in clinical remission or reduced inflammation, though noted improvements in patient-reported symptoms and quality of life. (Doeve et al. 2021) Both reviews relied on studies with small sample sizes, high potential for biases, and did not include several recently published studies. (Naftali et al. 2021; Coates et al. 2022) Three systematic reviews incorporating more recent studies collectively support that cannabis can improve quality of life in patients with Crohn’s disease, or ulcerative colitis, yet had conflicting results regarding cannabis impact on disease activity and remission. (Kumar 2024; Kumar et al. 2024; Kang et al. 2024) No recent reviews include non-interventional studies, examined patient reported outcomes beyond quality of life, such as pain or daily functioning, or focused on non-FDA approved cannabis products.

In the light of these conflicting findings, the inclusion of non-interventional studies, alongside evidence on additional patient reported outcomes from randomized clinical trials could provide valuable real-world insights into the effectiveness of cannabis in treating patients with IBD. Providing a comprehensive synthesis of current evidence is critical to quantify cannabis risk–benefit, especially in chronic use, among the growing population of IBD patients who are seeking symptom relief. (Lal et al. 2011; Velez-Santiago et al. 2023; Hilton Boon et al. 2022; Pratt et al. 2019) Therefore, we aimed to summarize and critically evaluate evidence from controlled non-interventional studies and controlled interventional trials on the effectiveness of THC-containing cannabis in patients with IBD.

## Methods

### Study design

This review followed the recommendations from the Cochrane Handbook for Systematic Reviews of interventions. (Higgins et al. 2019) Reporting of results are in alignment with the Preferred Reporting Items for Systematic Review and Meta-Analyses (PRISMA) statement. (Moher et al. 2010) The protocol is registered on PROSPERO (CRD42023411910), and no protocol deviations occurred during the conduct of the review (Goodin 2023).

### Search strategy

We developed separate search strategies for cannabis exposure and for the condition of interest (IBD and related conditions). All searches applied both search strategies, which combined keywords with controlled vocabulary constructed in consultation with subject matter experts (Additional File 1). We performed searches in February 2023 in PubMed, the Cochrane Library, APA Psych Info, and Embase. Literature searches limited results to publications from 2000 onwards to identify literature published since the 1999 Institute of Medicine’s comprehensive evidence review titled, Marijuana and Medicine (Institute of Medicine (US) 1999) and to English language. To ensure robustness and timeliness, the search strategy was rerun in PubMed and Embase to identify RCTs published before 2000 and newly published between February 2023 and February 2026.

### Management of search results

The Librarian de-duplicated the list of search hits, including citations and the accompanying abstracts, and imported them into the Covidence systematic literature review management system. (Covidence Systematic Review Software 2024) The use of Covidence as a systematic review management system can reduce inconsistencies in applying study exclusion criteria. (Kellermeyer et al. 2018; Macdonald et al. 2016).

### Screening procedures

All search results underwent title and abstract screening by two independent reviewers. Qualifying studies then underwent full text screening for inclusion by two independent reviewers, and any with conflicting assessments were reviewed by a third reviewer. We included studies if they were peer-reviewed, original research or systematic reviews/meta-analyses of original research examining the effectiveness of cannabis as a treatment modality; had the full text available; employed patient-level controlled designs; and provided a quantitative measure of effectiveness specifically related to the treatment of IBD (Crohn’s Disease and Ulcerative Colitis). We excluded studies if they were published before January 1, 2000, were not in English, included non-human research, examined cannabinoid products or synthetic cannabinoids approved by the US Food and Drug Administration (FDA) (i.e., Epidiolex [cannabidiol], Marinol/Syndros [dronabinol], and Cesamet [nabilone]), involved cannabis or cannabinoid formulations lacking delta-9-tetrahydrocannabinol (THC) or containing less than 0.3% THC on a dry weight basis (legal definition of hemp as defined in the Agriculture Improvement Act of 2018, Pub. L. 115–334, also known as 2018 Farm Bill), or employed topical marijuana formulations.

### Data extraction

Two abstractors independently extracted data from primary literature on study design, setting, population description including in- and exclusion criteria, sample size, covariates balanced (if any), exposure definition, outcome definition, and effect measures. Following initial abstraction, a third abstractor examined concordance of each element. SJ, AG, and AW, reviewed discordant abstractions and determined the characterization of each abstracted data element. The protocol included a pre-specified set of response options for study design type and setting, which abstractors used during data extraction. Systematic reviews were consulted solely to identify additional relevant primary studies.

Pre-specified primary outcomes of interest included induction or maintenance of remission (e.g., measured via the clinical disease activity index (CDAI), disease activity index (DAI), or Mayo endoscopic sub-score), and pain (e.g., measured via visual analog scales, abdominal pain index, and pain coping questions (PCQ)). Additional pre-specified outcomes of interest included quality of life (including general, e.g., 36-Item Short Form Survey (SF‑36) or disease-specific instruments, e.g., the short inflammatory bowel disease questionnaire (SIBDQ)); symptoms (e.g., four-point severity scale), and (physical) functioning, using validated tools such as the Functional Disability Inventory (FDI). The data extraction process for non-interventional studies captured all outcomes reported in included studies, regardless of whether the outcomes matched the pre-specified criteria.

### Risk of bias assessment

Two independent raters evaluated the risk of bias in non-interventional controlled studies using the Cochrane-endorsed “Risk of Bias in Non-Randomized Studies of Interventions” (ROBINS-I) checklist-style tool (Sterne et al. 2016) and evaluated RCTs for risk of bias with the Cochrane-endorsed “Risk of Bias in Randomized Trials version 2” (ROB2) checklist-style tool. (Sterne et al. 2019) The scores were summarized for each study and a third rater examined the two assessments for disagreements. In cases where two out of three raters did not achieve consensus, AG or AW independently evaluated the studies using the appropriate tool and determined the final classification. Results of the risk-of-bias assessment were visualized using robvis, a web application developed for displaying risk-of-bias evaluations (McGuinness 2021).

### Evidence synthesis and quality ratings

We described study characteristics separately for randomized (interventional) trials and non-interventional studies. Outcomes were reported based on outcome type, direction (improvement, worsening, no significant change, and not reported), and the count of studies reporting each specific outcome. Due to the small number of included studies assessing the same relevant outcome, the analysis did not compute meta-analytic summary estimates. If a study reported several *p*-values for an outcome as a result of using multiple comparison strategies (e.g., assessment of within and between group differences), the analysis prioritized them using the following hierarchical approach: 1. between group differences in changes in outcome from baseline to end of follow-up, 2. between group differences at end of follow-up if baseline values were not significantly different; 3. within group changes during follow-up if baseline values were significantly different. The analysis excluded effect estimates from non-interventional studies with serious or critical risk of bias ratings from the summary.

In accordance with the Cochrane-endorsed GRADE approach, the final evidence quality rating for each specific clinically relevant outcome (Higgins et al. 2019) included the following domains: certainty, precision, consistency, generalizability, and absence of publication bias. The author committee (SJ, AW, YW, SG, and AG) assessed rating via majority vote, per-domain-per-outcome and then overall rating per outcome, following committee review of abstracted data. Outcomes that were conceptually redundant with other measures—such as the Subjective Global Impression of Change, overall well-being, and general health,—were excluded from the evidence-quality assessment. We also omitted highly subjective outcomes that are particularly susceptible to measurement error, exemplified by the Physician Global Assessment of Illness Severity (PGAS) in comparison with the clinical disease-activity indices, specifically since PGAS was assessed as secondary outcome. Based upon this, self-reported changes in mood, memory, concentration, sleep, alertness, and general satisfaction were also excluded from the evidence-quality assessment.

## Results

**Fig. 1:**
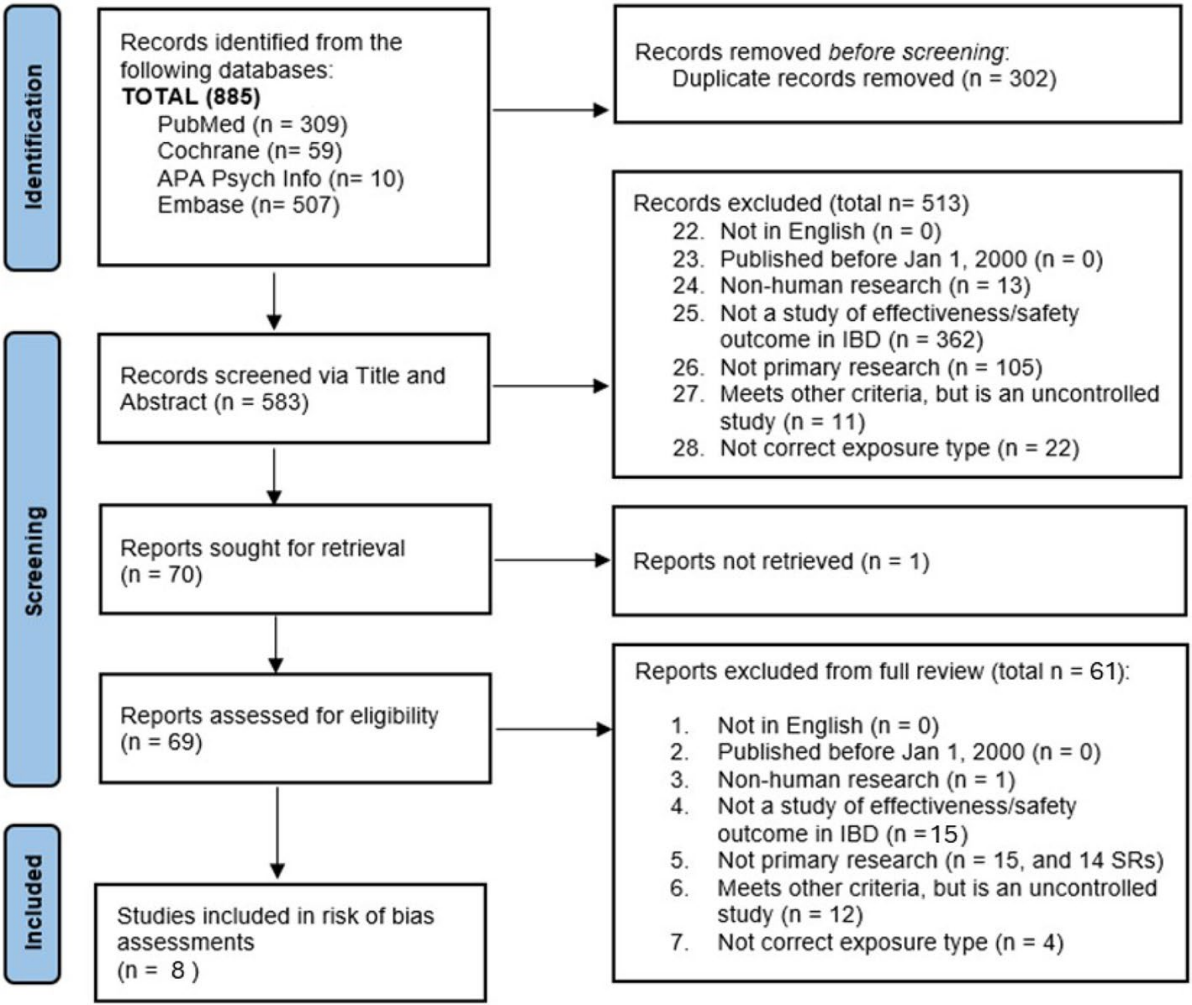
Flowchart of studies included in the systematic review. Study selection flow diagram for the systematic review, detailing records identified in four databases (*n* = 885), abstract screened (*n* = 583), assessed for eligibility (*n* = 69), and reasons for exclusion. Eight studies met inclusion criteria and were retained for risk of bias assessment

Across all databases the search identified 885 records; duplicate removal eliminated 302 duplicates (Fig. 1). Of the 583 articles screened, 8 studies met all inclusion criteria, underwent risk of bias assessment, and contributed to the evidence synthesis. The rerun of the search strategy in PubMed and Embase did not identify any additional eligible published RCTs.

### Characteristics of randomized clinical trials

All four RCTs used placebos (olive oil with chlorophyll, tobacco cigarettes, and capsules with excipients only) and enrolled small samples of adult IBD patients (*n* < 60) (Table 1). (Naftali et al. 2021, 2013; Irving et al. 2018) The treatment period ranged from 56 days (Naftali et al. 2021) to 70 days (Irving et al. 2018; Naftali et al. 2013). Studies focused on mild-to-moderate IBD, except for Naftali 2013, which included moderate-to-severe cases. Two studies (Naftali et al. 2021, 2013) evaluated cannabis flower with varying THC content (0.4% and 16% THC); Naftali (2021a) investigated oral CBD-rich oil containing 16% CBD and 4% THC; and the remaining two studies assessed oral capsules with 50 mg CBD per capsule and less than 4.7% THC. All studies defined disease activity or remission as the primary endpoint but used different scores and/or cut-offs (Crohn’s Disease Activity Index (CDAI) score, Lichtiger Score, and Mayo Score). More granular information of included RCTs are available in Additional File 1: Table S2, and Additional File 3.

**Table 1:** Characteristics of randomized controlled trials

First Author	Country	Control group	Treatment period (days)	Patients (Intervention/Control)	Patient population	Intervention	Primary outcome
Naftali 2021a	Israel	placebo (olive oil + chlorophyll)	56	30/26	-Adults, mild-to- moderate CD -excluded patients with pregnancy, UC, surgery during the study, known psychiatric diagnosis or addiction traits	-oral CBD rich oil (16% CBD/4% THC) −1 drop (8 mg CBD and 2 mg THC) twice daily -Max daily dose: 20 drops × 2	CDAI, score*, QoL SF-36 (score 0–100), Remission of disease (Endoscopic Disease Activity Index; SES-CD Score), Remission of disease (CRP [mg/dl]), Remission of disease (calprotectin [ug/g]), Clinical effect/Life impact (Improvement ≥ 30 points in QoL measured by SF-36), CD (abdominal pain), number of bowel movement per day, Weight [kg], Mood, Memory, Concentration, Sleep, Alertness, Daily function, Pain, Bloating, Nausea, Appetite, General well-being, General satisfaction, General effect on health, SES score (simple endoscope score), Adverse events
Naftali 2021b	Israel	placebo (cigarettes)	56	17/15	-Adults, mild-to-moderate UC, -excluded patients with pregnancy, severe UC, proctitis, psychiatric diagnosis or addiction traits	-inhaled flower (16% THC/0.1% CBD) -Start dose: Half cigarette (0.25 g) per day; -titrate to one cigarette (0.5 g) × 2/d	Lichtiger Score*, number of bowel movements per day, abdominal pain ≥ 2, QoL SF-36, Mayo endoscopic score, Self-report of side effects
Naftali 2013	Israel	placebo (cigarettes)	70	12/22	-Adults, moderate-to- severe CD -failed ≥ 1 medical treatment -stable under treatment -excluded patients with pregnancy, prior surgery, history of mental illness or addiction traits	-inhaled flower (0.4% THC) -One cigarette twice daily	Complete remission (CDAI score ≤ 150 after 8 weeks of treatment)*, Complete remission (CDAI score ≤ 150 after 8 weeks of treatment), response rate (defined as 100 point reduction of CDAI), QoL SF-36, side-effects
Irving 2018	UK	placebo (capsules with excipi-ents only)	70	29/31	-Adults, mild-to-moderate UC, -excluded patients with pregnancy, severe UC, proctitis, known psychiatric diagnosis or addiction traits	-oral Capsule (50 mg CBD per capsule, ≤ 4.7% THC) -Start dose: 1 capsule -Max daily dose: 5 capsules × 2	Remission, Mayo score ≤ 2*, physician global assessment of illness severity (PGAS) score, IBDQ, stool frequency, rectal bleeding, Mayo Total Score, Improvement in SGIC Questionnaire, Mayo Partial Score, adverse events (not including serious)

*Abbreviations*: *CDAI* Clinical Disease Activity Index, *CBD* Cannabidiol, *CD* Crohn’s disease, *CRP* C-reactive protein, *IBDQ* Inflammatory Bowel Disease Questionnaire, *PGAS* Physician Global Assessment of Illness Severity score, *QoL* Quality of Life, *SES* Simple Endoscopic Score, *SGIC* Subjective Global Impression of Change, *THC* Delta-9-tetrahydrocannabinol, *UC* Ulcerative colitis

^*^Indicates primary outcome

### Characteristics of non-interventional studies

All four identified non-interventional studies utilized US-based electronic healthcare data sources and cross-sectional designs (Table 2). (Coates et al. 2022; Mbachi et al. 2019; Desai et al. 2019) Studies included patients with Crohn’s disease, ulcerative colitis, or both (Additional File 4). One study assessed cannabis exposure via survey, (Coates et al. 2022) while all others used diagnosis codes to identify cannabis dependence or nondependent cannabis abuse. All studies used control groups of non-cannabis users, defined either based on absence of any relevant diagnosis codes or via survey questions. (Coates et al. 2022) Only one study defined a main outcome of interest (length of hospital stay for patients with Crohn’s disease). (Mbachi et al. 2019).

**Table 2:** Characteristics of included non-interventional studies

First Author		Data source (Country)	Study Design	Control Group	Patients Exposed vs. Control	Patient population	Exposure	Outcome assessed
Mbachi 2019	Nationwide Inpatient Sample (US)		Cross-Sectional	Non-User (absence of ICD codes)	298//39,504	-Hospitalized adult patients -Primary diagnosis of UC or Complication of UC + secondary Diagnosis Of UC -Excluded patients with substance use disorder	-Cannabis dependence (ICD9: 304.3, 304.3X) -Nondependent Cannabis Abuse (ICD9: 305.2X)	Length of stay, bowel obstruction, anemia, partial or total colectomy, blood transfusion, gastrointestinal bleeding, lower gastrointestinal endoscopy, upper gastrointestinal endoscopy
Desai, R. 2019	Nationwide Inpatient Sample (US)		Cross-Sectional	Non-User	4,199//258,079	-Adult patients admitted to the hospital -Diagnosis of CD or regional enteritis	-Cannabis dependence (ICD9: 304.3, 304.3X) -Nondependent Cannabis Abuse (ICD9: 305.20, 305.21, 305.22)	Anemia, hypovolemia, fluid and electrolyte disorders, active fistulizing disease, stricturing disease, intestinal obstruction, unspecified lower gastrointestinal hemorrhage, malnutrition, Clostridium difficile infection, colorectal cancer, small intestinal or colorectal resection, postoperative infection, blood transfusion, parenteral nutrition, length of stay
Mbachi 2019	Nationwide Inpatient Sample (US)		Cross-Sectional	Non-User (absence of ICD codes)	615//42,702	-Hospitalized adult patients -Primary diagnosis of CD, or secondary diagnosis + complication of CD -Excluded patients with substance use disorder	-Cannabis dependence (ICD9: 304.3X) -Nondependent Cannabis Abuse (ICD9: 305.2X)	Length of stay*,active fistulizing disease and intra-abdominal abscess, stricturing bowel disease, bowel obstruction, anemia, small bowel resection, partial or total colectomy, parenteral nutrition blood product transfusion
Coates 2022	IBD registry (US)		Cross-Sectional	Non-User (Survey question)	30/353	-Adult patients with a diagnosis of CD, UC, or IBD Colitis	-Survey Question	Abdominal pain, anxiety and/or depression, arthralgia, fatigue, gas, tenesmus, diarrhea, fecal urgency, rectal bleeding, current extraintestinal manifestations, dermatopathies, erythema nodosum, primary sclerosing cholangitis, uveitis

*Abbreviations*: *CD* Crohn’s disease, *UC* Ulcerative Colitis, *IBD* Inflammatory bowel disease, *ICD* International classification of disease

^*^Indicates primary outcome

### Risk of bias in randomized clinical trials

**Fig. 2:**
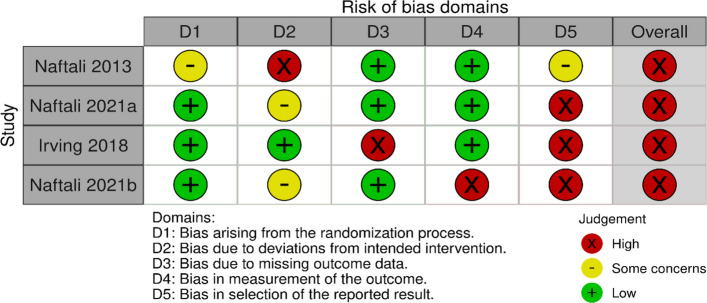
Risk of bias assessment for randomized controlled trials. Risk of bias assessment of the four randomized controlled trials included in this review, using the Cochrane RoB 2.0 tool across domains D1–D5 and overall judgement. Green circles indicate low risk of bias, yellow circles some concerns, and red circles high risk of bias

Reviewers assigned all RCTs an overall high risk of bias, primarily due to bias in the selection of reported results, which created mismatches between outcomes that were assessed according to the study protocol and those ultimately reported (Fig. 2). Evaluations identified bias due to deviations from the intended intervention as some concern in two studies and a high concern in one study, (Naftali et al. 2013) given observed protocol deviations.

### Risk of bias in non-interventional studies

**Fig. 3:**
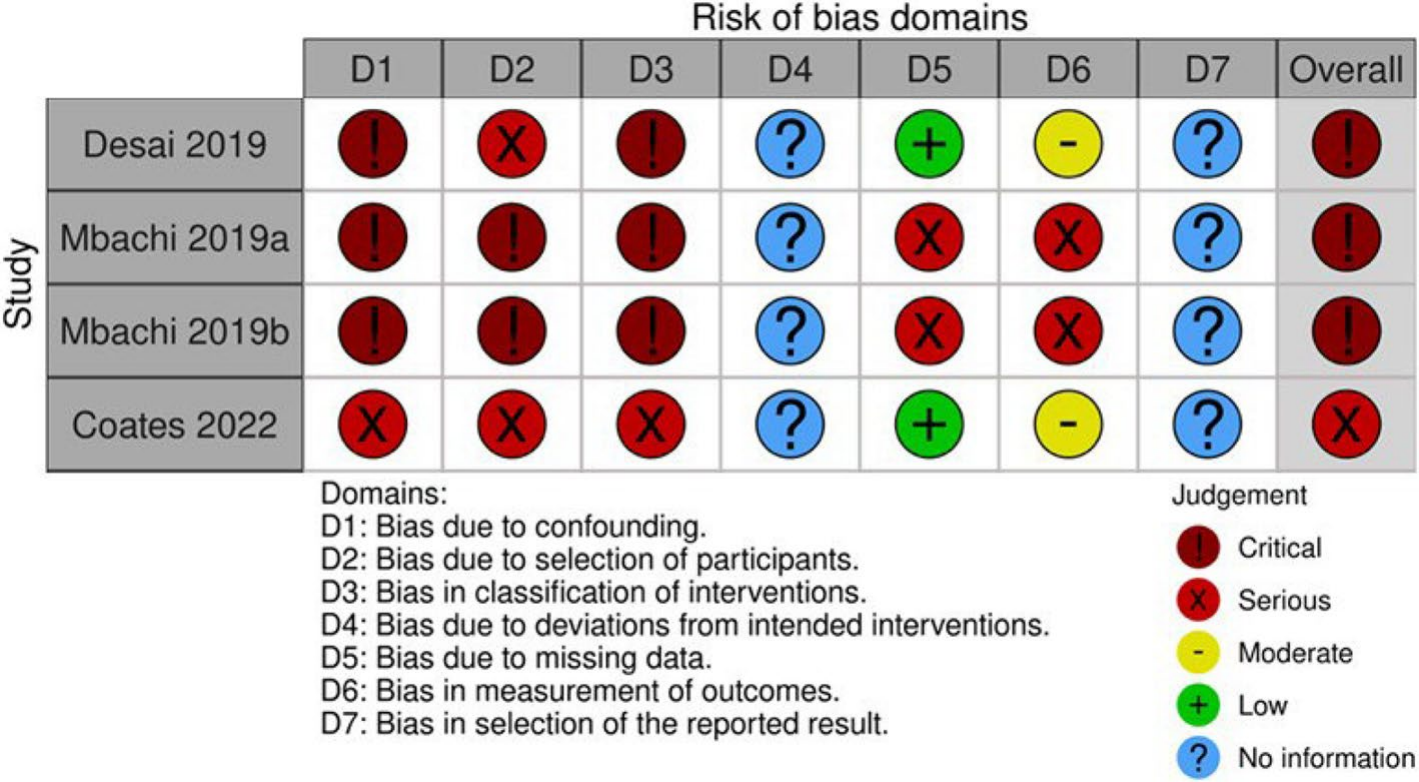
Risk of bias assessment for non-interventional studies. Risk of bias assessment of the four non-interventional studies included in this review, using the ROBINS-I tool across seven bias domains (D1–D7) and overall judgement. Green circles indicate low risk of bias, yellow moderate risk, red serious, dark red critical risk, and blue no information

Reviewers assigned three studies a critical level of bias, and one study (Coates et al. 2022) a serious level of bias (Fig. 3). Across studies, evaluations identified lower levels of concern for bias due to missing data. Assessments classified bias arising from participant selection and intervention classification as either critical or serious, reflecting reliance on diagnosis codes in administrative data indicating any cannabis use (e.g., Desai 2019, Mbachi 2019a, Mbachi 2019b) or self-reported cannabis use distinguishing current from non-current users (Coates 2022). Similarly, evaluations rated bias due to confounding as serious or critical in all non-interventional studies reviewed. Assessments of bias in outcome measurement categorized it as serious or moderate across studies.

### Qualitative evidence synthesis for randomized clinical trials

**Fig. 4:**
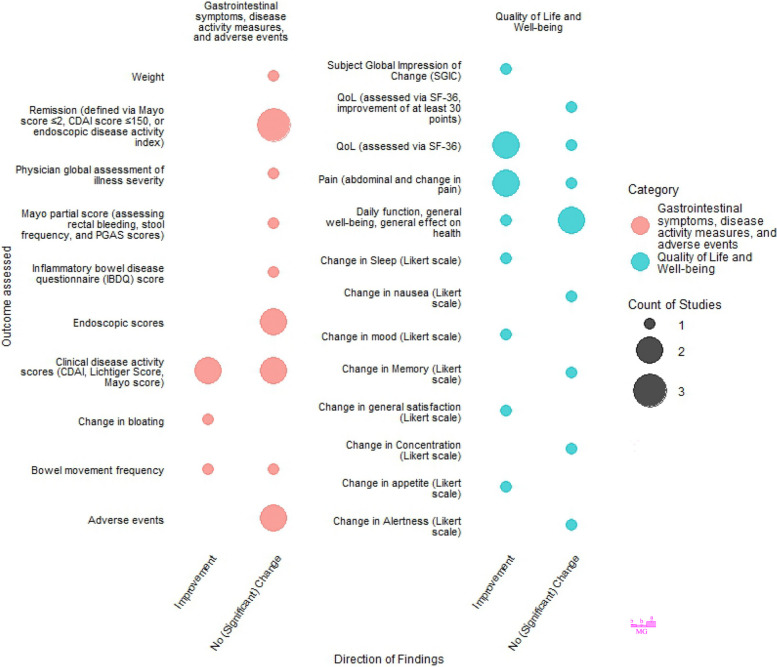
Direction of Findings Across Outcomes in RCTs of THC-Containing Cannabis Products for IBD. Bubble plot summarizing outcomes assessed in included RCTs. The x-axis shows the reported direction of findings (improvement vs no statistically significant change); the y-axis lists individual outcomes. Bubble size reflects the number of studies reporting each finding, and colors denote outcome domains (gastrointestinal symptoms/disease activity/adverse events vs quality of life and well-being). Abbreviations: c; QoL, quality of life; IBDQ, Inflammatory Bowel Disease Questionnaire; CDAI, Crohn Disease Activity Index; SF-36, 36-Item Short Form Health Survey

Each of these outcomes were evaluated once: sleep, appetite, bloating, general satisfaction, mood, (Naftali et al. 2021) and subject global impression of change; (Irving et al. 2018) cannabis exposure correlated with improvement in all compared to the control group (Fig. 4).

Conversely, the outcomes such as potentially treatment related adverse events, physician global assessment of illness severity, (Irving et al. 2018) remission, (Naftali et al. 2021, 2013; Irving et al. 2018) endoscopic scores, (Naftali et al. 2021) alertness, concentration, memory, nausea, (Naftali et al. 2021) disease-specific quality of life (IBDQ), Mayo partial score, (Irving et al. 2018) and quality of life (QoL as measured by an improvement of ^3^30 points on the SF-36) (Naftali et al. 2021) showed no significant changes. Among these, most appeared in a single assessment, except for adverse events and endoscopic scores, assessed twice each, and remission, assessed three times.

Mixed findings emerged for pain, clinical disease activity scores, (Naftali et al. 2021, 2013) bowel movements frequency, (Naftali et al. 2021; Irving et al. 2018) and QoL (via SF-36). Disease activity scores demonstrated improvement twice (Naftali et al. 2021, 2013) and no significant change twice. (Naftali et al. 2021; Irving et al. 2018) For QoL and pain (abdominal and change in pain), two studies reported improvement, (Naftali et al. 2021, 2013) while one found no significant change. (Naftali et al. 2021) For bowel movements frequency, improvement and no significant change was reported once each, and one study did not report statistical significance. (Irving et al. 2018) No study reported worsening of any assessed outcome. Although rectal bleeding was evaluated once, the study did not report a corresponding statistical test result to assess differences. (Irving et al. 2018) Notably, one trial reported both unadjusted and adjusted results for certain outcomes (see Additional File 3), with the latter accounting for baseline imbalances in age, gender, and illness duration. Therefore, the adjusted results were used in the analysis. (Naftali et al. 2021).

### Qualitative evidence synthesis for non-interventional studies

**Fig. 5:**
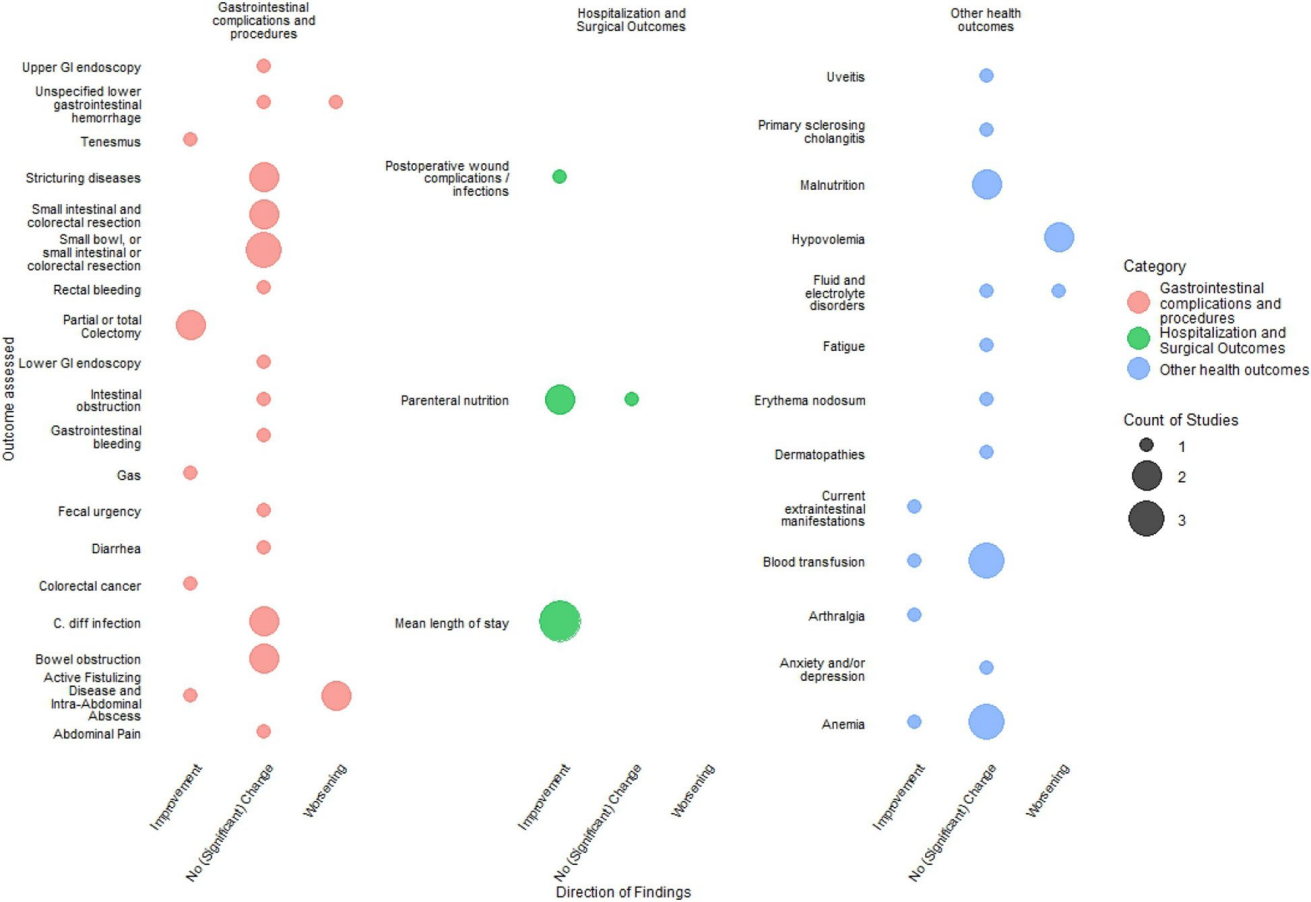
Direction of Findings Across Outcomes in Non-Interventional Studies of THC-Containing Cannabis Products for IBD. Bubble plot summarizing outcomes reported across included non-interventional studies of THC-containing cannabis products in inflammatory bowel disease. The x-axis shows the reported direction of findings (improvement, no statistically significant change, or worsening); the y-axis lists specific outcomes. Bubble size reflects the number of studies reporting each finding, and colors denote domains: gastrointestinal complications and procedures (red), hospitalization and surgical outcomes (green), and other health outcomes (blue). Abbreviations: GI, gastrointestinal; PSC, primary sclerosing cholangitis; C. diff, Clostridioides difficile

In non-interventional studies, patients defined as cannabis-exposed (often based on diagnostic codes for cannabis use, abuse, or dependence) compared to patients defined as not-cannabis exposed were associated with more favorable outcomes throughout all studies for arthralgia, (Coates et al. 2022) colorectal cancer, (Desai et al. 2019) current extraintestinal manifestations, gas, (Coates et al. 2022) length of stay, (Mbachi et al. 2019; Desai et al. 2019) partial or total colectomy, (Mbachi et al. 2019) postoperative wound complications/infections, (Desai et al. 2019) and tenesmus (Fig. 5). (Coates et al. 2022) Among these, length of stay appeared in four assessments, partial or total colectomy in two, and all others in a single assessment.

Several outcomes consistently showed no significant differences between cannabis-exposed and non-cannabis-exposed patients. These included abdominal pain, anxiety and/or depression, (Coates et al. 2022) bowel obstruction, (Mbachi et al. 2019) C. difficile infection, (Desai et al. 2019) dermatopathies, diarrhea, erythema nodosum, fatigue, fecal urgency, (Coates et al. 2022) gastrointestinal bleeding, (Mbachi et al. 2019; Desai et al. 2019) intestinal obstruction, (Desai et al. 2019) lower gastrointestinal tract endoscopy, (Mbachi et al. 2019) malnutrition, (Desai et al. 2019) primary sclerosing cholangitis, rectal bleeding, (Coates et al. 2022) small bowel or small intestinal or colorectal resection, (Mbachi et al. 2019; Desai et al. 2019), stricturing diseases, (Mbachi et al. 2019; Desai et al. 2019) upper gastrointestinal tract endoscopy, (Mbachi et al. 2019) and uveitis. (Coates et al. 2022) Small bowel or small intestinal or colorectal resection appeared in three assessments, while bowel obstruction, C. difficile infection, malnutrition, and stricturing diseases appeared twice each. All remaining outcomes in this category were assessed once.

Two assessments evaluated hypovolemia and showed less favorable outcomes in cannabis users compared to non-cannabis users in both. (Desai et al. 2019).

Results for remaining outcomes were inconsistent. Anemia appeared in four assessments, with one favoring cannabis exposed patients (Desai et al. 2019) and three showing no significant difference. (Mbachi et al. 2019; Desai et al. 2019) Blood transfusion demonstrated a similar pattern. (Mbachi et al. 2019; Desai et al. 2019) Parenteral nutrition showed beneficial outcomes for cannabis exposed patients in two assessments (Mbachi et al. 2019; Desai et al. 2019) and one reporting no significant change. (Desai et al. 2019) Active fistulizing disease and intra-abdominal abscess were associated with more favorable outcomes once (Mbachi et al. 2019) and worse outcomes twice. (Desai et al. 2019) Cannabis exposure was associated with no significant difference in one assessment and worsening in another, compared to non-cannabis exposed patients, for the outcomes of fluid and electrolyte disorders, and unspecified lower gastrointestinal. (Desai et al. 2019).

### Evidence ratings

Evaluations identified all non-interventional studies as having a high or critical risk of bias; consequently, these studies were not included in quality of evidence ratings. Among outcomes evaluated in RCTs, quality of evidence ratings varied (Table 3).

**Table 3:** Summary of quality of evidence ratings by IBD outcome, randomized clinical trials

**Outcome**	**Quality Domain**	**Naftali 2013** (Naftali et al. 2013)	**Naftali 2021a** (Naftali et al. 2021)	**Naftali 2021b** (Naftali et al. 2021)	**Irving 2018** (Irving et al. 2018)	**Overall Rating across studies**
Clinical Disease Activity Indexes (CDAI/Lichtiger/Mayo Score)	Certainty	Moderate Concern	Moderate Concern	Moderate Concern	Moderate Concern	Moderate Concern
	Imprecision	Moderate Concern	Low Concern	Low Concern	High Concern	Moderate Concern
	Inconsistency	Moderate Concern				
	Generalizability	Moderate Concern	Moderate Concern	Moderate Concern	High Concern	Moderate Concern
	Publication Bias	Moderate Concern				
	Overall Quality of Evidence Rating	Moderate Quality				
Daily Function, General Well-Being, General Effect on Health	Certainty	NR	High Concern	NR	NR	High Concern
	Imprecision	NR	Moderate Concern	NR	NR	Moderate Concern
	Inconsistency	Unable to Rate				
	Generalizability	NR	Moderate Concern	NR	NR	Moderate Concern
	Publication Bias	Moderate Concern				
	Overall Quality of Evidence Rating	Low Quality				
Quality of Life	Certainty	Moderate Concern	Moderate Concern	Moderate Concern	NR	Moderate Concern
	Imprecision	Low concern	Low Concern	Low Concern	NR	Low Concern
	Inconsistency	Moderate Concern				
	Generalizability	Moderate Concern	Moderate Concern	Moderate Concern	NR	Moderate Concern
	Publication Bias	Moderate concern				
	Overall Quality of Evidence Rating	Moderate Quality				
Pain	Certainty	NR	Moderate Concern	Moderate Concern	NR	Moderate Concern
	Imprecision	NR	Moderate concern	Low concern	NR	Moderate Concern
	Inconsistency	Moderate Concern				
	Generalizability	NR	Moderate Concern	Moderate Concern	NR	Moderate Concern
	Publication Bias	Moderate Concern				
	Overall Quality of Evidence Rating	Moderate Quality				
Remission	Certainty	Moderate Concern	High Concern	Moderate Concern	NR	Moderate Concern
	Imprecision	Moderate Concern	Moderate Concern	Moderate Concern	NR	Moderate concern
	Inconsistency	Low Concern				
	Generalizability	Moderate Concern	Moderate Concern	Moderate Concern	NR	Moderate Concern
	Publication Bias	Low Concern				
	Overall Quality of Evidence Rating	Moderate Quality				
Number of bowel movements/stool frequency	Certainty	NR	Serious Concern	Serious Concern	Serious Concern	Serious Concern
	Imprecision	NR	Moderate Concern	Low Concern	Unable to report	Moderate Concern
	Inconsistency	Moderate Concern				
	Generalizability	NR	Moderate Concern	Moderate Concern	Moderate Concern	Moderate Concern
	Publication Bias	Moderate Concern				
	Overall Quality of Evidence Rating	Low Quality				
Rectal bleeding	Certainty	NR	NR	NR	Serious Concern	Serious Concern
	Imprecision	NR	NR	NR	Serious Concern	Serious Concern
	Inconsistency	Unable to Rate				
	Generalizability	NR	NR	NR	Moderate Concern	Moderate Concern
	Publication Bias	Low Concern				
	Overall Quality of Evidence Rating	Low Quality				
Weight	Certainty	NR	Low Concern	NR	NR	Low Concern
	Imprecision	NR	Low Concern	NR	NR	Low Concern
	Inconsistency	Unable to Rate				
	Generalizability	NR	Moderate Concern	NR	NR	Moderate Concern
	Publication Bias	Low Concern				
	Overall Quality of Evidence Rating	High Quality				
Disease-specific QoL (IBDQ)	Certainty	NR	NR	NR	Moderate concern	Moderate Concern
	Imprecision	NR	NR	NR	Moderate Concern	Moderate Concern
	Inconsistency	Unable to Rate				
	Generalizability	NR	NR	NR	Moderate concern	Moderate Concern
	Publication Bias	Low Concern				
	Overall Quality of Evidence Rating	Moderate Quality				
Bloating	Certainty	NR	High concern	NR	NR	High Concern
	Imprecision	NR	Moderate concern	NR	NR	Moderate Concern
	Inconsistency	Unable to Rate				
	Generalizability	NR	Moderate Concern	NR	NR	Moderate Concern
	Publication Bias	Moderate Concern				
	Overall Quality of Evidence Rating	Low Quality				
Nausea	Certainty	NR	Serious concern	NR	NR	High Concern
	Imprecision	NR	Moderate concern	NR	NR	Moderate Concern
	Inconsistency	Unable to Rate				
	Generalizability	NR	Moderate Concern	NR	NR	Moderate Concern
	Publication Bias	Moderate Concern				
	Overall Quality of Evidence Rating	Low Quality				
Appetite	Certainty	NR	High concern	NR	NR	High Concern
	Imprecision	NR	Moderate concern	NR	NR	Moderate Concern
	Inconsistency	Unable to Rate				
	Generalizability	NR	Moderate Concern	NR	NR	Moderate Concern
	Publication Bias	Moderate Concern				
	Overall Quality of Evidence Rating	Low Quality				
Endoscopy Assessment (simple endoscope score; mayo endoscopic score)	Certainty	NR	Moderate Concern	Moderate Concern	NR	Moderate Concern
	Imprecision	NR	Moderate Concern	Moderate Concern	NR	Moderate Concern
	Inconsistency	Low Concern				
	Generalizability	NR	Moderate Concern	Moderate Concern	NR	Moderate Concern
	Publication Bias	Moderate Concern				
	Overall Quality of Evidence Rating	Moderate Quality				

*Abbreviations*: *IBDQ* Inflammatory Bowel Disease Questionnaire, *NR* Not reported

Low-quality evidence supported patient-reported daily functioning, general well-being, overall health effects, frequency of bowel movements, rectal bleeding, bloating, nausea, and appetite (Table 3). Moderate-quality evidence characterized clinical disease activity indices (CDAI), QoL, pain, remission status, disease-specific QoL, and endoscopic assessments, while high-quality evidence supported weight.

Several methodological concerns affected the certainty of findings in RCTs. For all self-reported outcomes (e.g., bowel movements, rectal bleeding, nausea, daily-functioning, general well-being, and general effect on health), serious concerns arose due to the partial lack of blinding arising from recognizable psychotropic effects. For bloating, nausea, and appetite, additional methodological concerns arose from exclusive post-intervention assessments, which led to high concerns regarding the certainty of the evidence. In contrast, for CDAI, QoL, pain, remission, disease-specific QoL, and endoscopic evaluations relied on less subjective and/or relied on standardized assessment tools, resulting in moderate (rather than high or serious) concerns about certainty – partly due to selective outcome reporting in some trials (e.g., CDAI). Objective measurement of weight contributed to low concern regarding certainty.

Evaluation classified imprecision in study findings as serious for rectal bleeding, primarily due to wide confidence intervals. Analyses identified low concern about imprecision for quality of life and weight, where studies reported narrow confidence intervals. For most other outcomes, statistical power varied markedly and baseline and post-intervention values (e.g., bowel movements) were heterogenous in many, though not all, studies, generating moderate levels of concern regarding precision.

Analyses identified moderate inconsistency for CDAI, quality of life, pain, and number of bowel movements, given variations in the direction of findings across studies. However, studies assessing remission and endoscopic findings displayed consistent results across trials, yielding low concern regarding inconsistency. Outcomes evaluated in only a single study could not be formally appraised for inconsistency.

Assessments assigned moderate concern regarding generalizability for all outcomes. This primarily stemmed from the focus on mild-to-moderate disease severity, the requirement for stable doses of certain medications (e.g., corticosteroids, thiopurines, methotrexate, and anti–TNF-α), the exclusion of participants with pre-existing psychiatric or addiction diagnoses, and notable attrition in select trials (e.g., Irving et al.).

Finally, moderate concern regarding publication bias has been noted for CDAI, patient-reported daily functioning, general well-being and overall health effects, quality of life, pain, number of bowel movements, bloating, nausea, appetite, and endoscopic assessments, owing to discrepancies between reported outcomes in manuscripts versus registered protocols. For all other outcomes, publication bias was of low concern, given the absence of such discrepancies.

## Discussion

In our systematic review of studies investigating THC-containing cannabis products for the treatment of IBD, we identified four RCTs and four non-interventional studies that met inclusion criteria, constituting the most up to date and inclusive synthesis on this topic. Several noteworthy findings emerged. First, among interventional studies and IBD outcomes assessed more than once, no single outcome demonstrated consistent improvement with cannabis use. Remission, examined in three trials, showed no significant differences between cannabis and control groups, suggesting with moderate confidence that cannabis may not enhance remission rates in patients with IBD. The moderate evidence quality of the heterogenous findings among studies examining disease activity scores and QoL indicate that evidence is insufficient to conclude whether cannabis might reduce disease activity scores and improve QoL in patients with IBD. While some outcomes assessed once demonstrated improvement (e.g., bloating and appetite), most carried low-quality evidence ratings, leading to high uncertainty in derived effect estimates. Weight change, assessed in a single small trial, was the only outcome rated as high-quality evidence and showed no effect of cannabis, yet the study might have been underpowered to capture small weight changes.

A second noteworthy finding was the incorporation of results from four recently published non-interventional studies with mixed findings regarding cannabis exposure on various IBD outcomes. Though, critical methodological limitations undermined confidence in their estimates for relevant clinical outcomes, hindering potential to draw sound conclusions based upon their findings, and the ability to provide robust actionable evidence in this field of research. We note that methodological constraints – including not only limitations in exposure measurement but also the heterogeneity and partial unblinding inherent to the cannabis interventions — reduced the quality of evidence across both interventional and non-interventional research. In light of these findings, this review identified no conclusive evidence of moderate or higher certainty supporting the effectiveness of THC-containing cannabis products in patients with inflammatory bowel disease. Accordingly, the current evidence does not support the use of these products as a substitute for approved therapies with established efficacy in reducing inflammation and improving symptoms.

Placing our findings in the context of previous systematic reviews reveals consensus for some outcomes and notable divergences for other IBD outcomes. Regarding clinical remission, our results concur with earlier analyses showing no consistent benefit of cannabis, a pattern similarly reflected in inflammatory biomarker findings. (Doeve et al. 2021; Kumar et al. 2024) QoL outcomes mostly align with earlier meta-analyses documenting improvements or mixed effects, though discrepancies may arise from our emphasis on adjusted analyses, which prior meta-analyses did not consider (e.g., preferred unadjusted trial results), and our exclusion of one study reporting in-vitro findings. (Doeve et al. 2021; Kumar 2024; Kang et al. 2024) We did not identify consistent improvement in disease activity scores, which is a departure from prior reviews. This may be attributable to our inclusion of more recent data, our inclusion of adjusted results, and consideration of different disease activity scoring systems under a single outcome type, rather than separating by disease entity or score type. (Doeve et al. 2021; Kumar et al. 2024; Kang et al. 2024; Vinci et al. 2022).

Confirming earlier systematic review findings, we observed no significant improvement in endoscopic outcomes after the addition of more recent studies. (Kang et al. 2024) Additional findings for clinical symptoms—such as nausea, appetite, and general well-being—partially differs from a previous meta-analysis, likely due to our inclusion of more recent studies. (Doeve et al. 2021) The modest improvement in bowel movements for ulcerative colitis reported by previous studies aligns with our own observations if separating our results between ulcerative colitis and Crohn’s disease. (Kumar 2024) In contrast, however, a previous meta-analysis suggested that cannabis exposure might provide pain relief; though, after including more recent studies and removing critically high risk of bias non-interventional studies, we did not consistently identify an improvement in pain among cannabis users when compared to controls. (Doeve et al. 2021).

Methodological challenges underscored significant barriers to deriving reliable effect estimates. In the non-interventional studies, severe risk of bias arose from insufficient control of confounders, including the absence of detailed data on concomitant medication use. Exposure misclassification further hampered interpretability, as the included studies relied on self-reported “ever” cannabis use or on diagnosis codes for cannabis dependence and cannabis use disorder, preventing an accurate quantification of current (acute) cannabis exposures, quantities of exposure, use duration and patterns of use, use modes, or cumulative cannabis exposures. Cross-sectional designs, which do not adequately capture the temporal sequence of exposure, outcome, and confounders, also precluded causal inferences in non-interventional studies. To address these issues, future non-interventional investigations need to adopt robust cannabis exposure measurement strategies as well as causal inference frameworks—such as target trial emulation—and integrate data sources that permit precise longitudinal exposure assessments. (Hernán et al. 2022; MMJ Outcomes 2024).

Included RCTs exhibited lower risk of bias, but limitations such as small sample sizes, reporting inconsistencies due to protocol deviations, challenges with blinding, and limited generalizability constrained the overall quality of evidence. Partial unblinding contributed to higher participant dropout rates across study arms, leading to issues with differential attrition in RCTs, and increased uncertainty in effect estimates specifically for subjective endpoints like pain and overall well-being. Three of the four RCTs deviated from prespecified protocols in terms of outcomes reported and analysis strategies, which could affect the transparency of reported findings. We observed limited generalizability since most trials did not include patients with moderate-to-severe IBD or complex comorbidities. Furthermore, the dosage and composition of cannabis interventions administered in trials often diverged from cannabis exposures in real-world settings, particularly in terms of THC content and frequency of use (e.g., THC content among medicinal and recreational programs is on average higher than 19%, whereas in included studies the THC content ranged from 0.6% to 16% THC). (Cash et al. 2020; Jugl et al. 2023) While the potential rescheduling of cannabis to a less restrictive category could reduce barriers to research and enable larger, more pragmatic RCTs, future studies also need to account for methodological challenges such as blinding in the presence of THC’s psychoactive effect. Ultimately, addressing these methodological shortcomings in both non-interventional and randomized research is imperative for a more definitive understanding of cannabis’s potential as a therapeutic in IBD.

Key strengths of this systematic review lie in its comprehensive search strategy, which encompassed both randomized trials and non-interventional studies, and thorough risk-of-bias and quality-of-evidence assessments. However, several limitations must be acknowledged. First, because few studies met inclusion criteria, our qualitative evidence synthesis pooled findings from heterogeneous populations, interventions, and control groups, prohibiting us from further exploring the effect of specific cannabis products (e.g., products with the same THC content, or the same cannabis product type) on reported outcomes or differences in effect sizes across specific subsets of patients. The preponderance of studies originating from the same research group raises questions regarding external validity, underscoring the need for replication by other investigators. High risks of bias resulted in substantial uncertainty and reduced quality of evidence for many outcomes. In addition, we opted not to incorporate findings from highly biased non-interventional studies into our quality-of-evidence ratings to avoid reliance upon very uncertain effect sizes. Moreover, although we excluded studies that solely focused on federally legal hemp or FDA-approved cannabinoid products—as they are not classified under Schedule I of the Controlled Substances Act (CSA)—in all but one non-interventional study, it was not feasible to distinguish between botanical cannabis, synthetic cannabinoids, and hemp due to the administrative nature of the data. Moreover, we did not evaluate FDA-approved or synthetic cannabinoid medicines (e.g., Epidiolex or dronabinol, and synthetic THC analogues such as nabilone). Because these products typically contain one cannabinoid (or a small, fixed set), whereas botanical cannabis comprises > 100 phytocannabinoids and hundreds of additional constituents (e.g., terpenes), our findings from cannabis products that contained more than one cannabinoid may not be generalizable to pharmaceutical cannabinoids. (André et al. 2024; Christensen et al. 2023) Moreover, since our search concluded in February 2023, we did search for any newly published RCTs between February 2023 and February 2026, which did not yield any new RCT, confirming timeliness of this review (French et al. 2005). Finally, the wide array of heterogenous outcome measures precluded the generation of meta-analytic summary estimates; instead, our ordinal summaries capture adjusted analyses and provide a useful framework to guide future research and policy. However, a limitation of this approach is that this synthesis relies largely on *p*-values, which can obscure clinically relevant effects when studies are heterogeneous and variably powered, ignores the magnitude and direction of observed effects, and invites misinterpretation when multiple comparisons are made.

## Conclusion

In conclusion, this systematic review did not identify high-quality evidence for a positive effect of THC-containing cannabis products in the treatment of IBD; yet, we identified high-quality evidence from one study suggesting that cannabis does not affect weight significantly in these patients. While bloating and appetite appeared to improve following cannabis use, the quality of evidence supporting these findings was low. In addition, analyses found no consistent or definitive effects on clinical disease activity, quality of life, pain, remission, IBD-specific quality of life, or endoscopic outcomes, with the supporting evidence for these null or inconclusive findings ranging from low to moderate in quality. Taken together, these findings do not support changes in clinical guidance related to THC-containing cannabis products for IBD. The evidence quality underscores the persistent methodological challenges existing in research on cannabis and its therapeutic utility in IBD, resulting in high degrees of uncertainty. Future investigations must prioritize rigorous cannabis exposure measurement strategies (e.g., longitudinal dispensing data rather than self-reported use for non-interventional studies), robust study designs (e.g., target trial emulation framework for non-interventional studies), standardized outcomes, and transparent, protocol-compliant reporting to clarify the role of cannabis as a potential treatment for IBD.

## Supplementary Information

Additional file 1. Supplemental methods (search strategy) and Table S2: Effect estimates and outcome data from randomized controlled trials informing quality-of-evidence ratings.

Additional file 2. Full Search Strings used across databases.

Additional file 3. Extracted data from included randomized clinical trials.

Additional file 4. Extracted data from included non-interventional studies.

## Data Availability

The protocol for this review describes data sources and analytic methods and was pre-registered via PROSPERO (available from: [CRD42023411910](https:/www.crd.york.ac.uk/prospero/display_record.php?ID=CRD42023411910)). All data and materials used in qualitative synthesis are available within supplemental materials.

## Abbreviations

CBR: Cannabinoid receptor
CDAI: Crohn’s Disease Activity Index
C. dif: Clostridioides difficile
CSA: Controlled Substances Act (US)
FAAH: Fatty acid amide hydrolase
FDA: U.S. Food and Drug Administration
GI: Gastrointestinal
IBD: Inflammatory bowel disease
IBDQ: Inflammatory Bowel Disease Questionnaire
PSC: Primary sclerosing cholangitis
QoL: Quality of life
RCT: Randomized clinical trial
SF-36: 36-Item Short Form Health Survey
SGIC: Subject Global Impression of Change
THC: Delta-9-tetrahydrocannabinol
TRPV1: Transient receptor potential vanilloid 1
