# Supplementary material for: Effectiveness of THC-containing cannabis for inflammatory bowel disease: a systematic review
Source: J Cannabis Res. 2026 Jun 3;8:92. doi: 10.1186/s42238-026-00456-2 (PMC13455333; doi:10.1186/s42238-026-00456-2)
Supplement: Supplementary file 1 [file 42238_2026_456_MOESM1_ESM.docx]

**Additional File 1**

Table of Contents

[Supplemental Methods	2](#_Toc214366047)

[Table S2. Effect Estimates and Outcome Data from RCTs Informing Quality of Evidence Ratings	3](#_Toc214366048)

[References	6](#_Toc214366049)

# Supplemental Methods

*Search Strategy*

We developed separate search strategies for cannabis exposure and for the condition of interest (IBD and related conditions). Keywords and controlled vocabulary constructed in consultation with subject matter experts were sourced from preliminary review as per citations summarized in Table S1. Two concepts were used in combination for searches, consisting of a concept for “cannabis” AND a concept for “Inflammatory bowel disease”. All databases (PubMed, Cochrane, Medline, Embase) deployed both search concepts. The full search strings are available in the Additional File 2.

Table S1: Keyword and Controlled Vocabulary Sources

| **Source of Keywords** | **Keyword (or MeSH, or Other Controlled Vocabulary)** | **Database(s) where sourced keywords were applied in previous reviews** |
| --- | --- | --- |
| **“Cannabis” Concept** | | |
| NASEM(1) | Cannabinoids; Cannabinoid; Cannabis; Marijuana; Marihuana; Tetrahydrocannabinol; THC; Cannabidiol; synthetic cannabinoid; SCB; Medical Marijuana; Medicinal Cannabis; Marijuana Treatment; Medical Cannabis; Cannabinol; Spice; spices; K2 | PubMed, Cochrane |
| Psych Info | Hashish | APA Psych Info |
| **“Inflammatory bowel disease” Concept** | | |
| NASEM(1) | Crohn Disease; crohn; crohns; Crohn's Enteritis; Regional Enteritis; Granulomatous Enteritis; Ileocolitis; Granulomatous Colitis; Terminal Ileitis; Regional Ileitis | PubMed |
| Norton, et. al. 2017(2) | Ulcerative Colitis; Colitis; Crohn Disease; Crohn*; Inflammatory Bowel Disease; IBD | Medline, Embase, PsyCInfo |
| Kafil, et al. 2018 (3) | Crohn disease; Crohn; IBD; Inflammatory bowel disease; Coon disease;  CUD | Medline, EMBASE, PsycCInfo |
| PubMed [MeSH] | "Colitis, Ulcerative"[Mesh]; "Colitis"[Mesh]; "Crohn Disease"[Mesh]; "Inflammatory Bowel Diseases"[Mesh] | PubMed |
| Embase [Emtree] | 'ulcerative colitis'/exp; 'colitis'/exp; 'Crohn disease'/exp; 'inflammatory bowel disease'/exp | Embase |
| Psych Info | DE "Ulcerative Colitis"; DE "Colitis" | APA Psych Info |

We performed searches in February 2023 in PubMed, the Cochrane Library, APA Psych Info, and Embase. Literature searches limited results to publications from 2000 onwards to identify literature published since the 1999 Institute of Medicine’s comprehensive evidence review titled, Marijuana and Medicine(4) and to English language.

# Table S2. Effect Estimates and Outcome Data from RCTs Informing Quality of Evidence Ratings

| **Study ID (Author Publication Year)** | **Outcome Measure Definition** | **Effect Estimate (95% CI or other measure of precision)** | **p-values** |
| --- | --- | --- | --- |
| **Clinical disease activity measures** | | | |
| **Naftali 2013** (5) | CDAI, score | I: 330±105 to 152±109//C: 373±94 to 306±143 | Difference in changes of the outcome between both groups p <0.05 |
| **Naftali 2021a** (6) | CDAI, score | I: visit one 282 [243–342] visit three 166 [82–226]// C: 264 [234–320] 237 [121–271] | Difference in the outcome between both groups at the end of study period:  -p=0.072, adjusted |
| **Naftali 2021b**(7) | Lichtiger Score (score 0-21) | I: 10.9 (IQR 9–14) to 5 (IQR 1–7) // C: 11 (IQR 9–13) to 8 (IQR 7–10) | Difference in the outcome between both groups at the end of study period:  -p=-0.006 |
| **Irving 2018**(8) | Mayo Total Score (range from 0 to 12 with higher scores indicating more severe disease) | I: 6.3 (1.73) to 3.0 (2.25) // C: 7.0 (2.01) to 4.9 (3.22) | Difference in changes of the outcome between both groups (90 % CI)  -ITT: p=0.14 |
| **Quality of Life (assessed via SF-36)** | | | |
| **Naftali 2013** (5) | QoL SF-36 (score 0-100) | I: 68 to 86 // C: 71 to 79 | Difference in changes of the outcome between both groups:  -p=0.04 |
| **Naftali 2021a**(6) | QoL SF-36 (score 0-100) | I: visit one 74 [65–87] visit three 91 [85–102]// C: 74 [57–82] 75 [69–88] | Difference in the outcome between both groups at the end of study period:  -p=0.143, adjusted |
| **Naftali 2021b** (7) | QoL SF-36 (score 0-100) | I: 77±4 to 98±20 // C:78±3 to 78±17 | Difference in the outcome between both groups at the end of study period:  -p=0.026 |
| **Pain** | | | |
| **Naftali 2021a** (6) | Pain (1-7 Likert scale (1=great improvement, 7=severe deterioration)) | I: 2.0±0.91// C: 3.38±1.35 | Difference in the outcome between both groups at the end of study period:  -p=0.002 |
|  | CD (abdominal pain) | I: 2 [1.25–2] to 1 [0–2] // C: 2 [1.75–2] to 2 [0–2] | Difference in the outcome between both groups at the end of study period:  -p=0.078, adjusted |
| **Naftali 2021b**(7) | abdominal pain ≥ 2 | I: 10 (59%) to 1 (6%) // C: 9 (60%) to 8 (55%), | Difference in the outcome between both groups at the end of study period:  -p=0.04 |
| **Remission** | | | |
| **Naftali 2013**(5) | Complete remission (CDAI score ≤ 150 after 8 weeks of treatment) | I: 5/11 (45%)//C: 1/10 (10 %) | Difference in the outcome between both groups at the end of study period:  -p=.43 |
| **Naftali 2021a**(6) | Remission of disease (Simple endoscopic score) | I: 10 [7–14] to 7 [4–14] // C: 11 [7–14] to 8 [4–12] | Difference in the outcome between both groups at the end of study period:  -p=0.185, adjusted |
|  | Remission of disease (CRP [mg/dl]) | I: 1.4 [0.4–2.7] to 1.7 [0.4–3.8] // C: 1.3 [0.2–2.2] to 1.5 [0.5–3] | Difference in the outcome between both groups at the end of study period:  -p=0.54, adjusted |
|  | Remission of disease (calprotectin [ug/g]) | I: 139 [64–300] to 112 [65–300] // C: 112 [50–185] to 117 [50–300] | Difference in the outcome between both groups at the end of study period:  -p=0.13, adjusted |
| **Irving 2018**(8) | Remission, Mayo score ≤ 2 | ITT:  I: 8 (27.6 %) / C: 8 (25.8 %) | Difference in changes of the outcome between both groups (90 % CI):  -p=0.753 |
| **Bowel Movement Frequency** | | | |
| **Naftali 2021a**(6) | number of bowel movement per day | I: 5 [3–7] to 2.5 [1–4] // C: 5 [3–8] to 3 [1.5–7.5] | Difference in the outcome between both groups at the end of study period:  -p=0.770, adjusted |
| **Naftali 2021b**(7) | number of bowel movements per day | I: 2.6 (IQR 2–4) to 1 (IQR 0–1 // C: 2.6 (IQR 2–4) to 2 (IQR 2–3) | Difference in the outcome between both groups at the end of study period:  -p=0.006 |
| **Irving 2018**(8) | stool frequency (NRS 0-3: 0 = normal number of stools, 3 = five or more stools more than normal) | I: 1.50 (0.937) to 1.05 (0.930) / C: 1.89 (0.814) to 1.46 (0.888) | Not reported |
| **Endoscopic Scores** | | | |
| **Naftali 2021a**(6) | SES score (simple endoscope score) | I: visit one 10 [7–14] visit three 7 [4–14]// C: 11 [7–14] 8 [4–12] | Difference in the outcome between both groups at the end of study period:  -p=0.185, adjusted |
| **Naftali 2021b**(7) | Mayo endoscopic score (score 0-12) | I: 2.13±1 to 1.25±2 // C: 2.15±1 to 1.69±1 | Difference in the outcome between both groups at the end of study period:  -p=0.374 |
| **Daily function, general well-being, general effect on health** | | | |
| **Naftali 2021a**(6) | Daily function (1-7 Likert scale (1=great improvement, 7=severe deterioration)) | I: 3.3±1.49 // C:3.86±0.79 | Difference in the outcome between both groups:  -p=0.22 |
|  | General well-being (1-7 likert scale (1=great improvement, 7=severe deterioration)) | I: 2.35±1.04 // C: 3.25±1.02 | Difference in the outcome between both groups:  -p=0.49 |
|  | General effect on health (1-7 likert scale (1=great improvement, 7=severe deterioration)) | I: 3.15±1.2 // C:3.8±1.4 | Difference in the outcome between both groups:  -p=0.007 |
| **Rectal bleeding** | | | |
| **Irving 2018**(8) | rectal bleeding (NRS 0-3: 0 = no blood seen, 3 = blood alone passes) | I: 0.96 (0.829) to 0.48 (0.711) / C: 1.19 (0.816) to 0.84 (0.863) | Not reported (ITT) |
| **Weight** | | | |
| **Naftali 2021a**(6) | Weight [kg] | I: 62 [56–77] to 62 [55–74] // C: 63 [52–78] to 64 [51–78] | Difference in the outcome between both groups at the end of study period:  -p=0.57, adjusted |
| **Disease-specific QoL (IBDQ)** | | | |
| **Irving 2018**(8) | IBDQ (Total score range: 32 to 224 points) | I: 138.5 (32.37) to 164.2 (29.13) / C: 129.1 (38.02) to 146.8 (47.50) (ITT) | Difference in the outcome between both groups:  -P-Value not reported (ITT), but reported as no significant change |
| **Bloating** | | | |
| **Naftali 2021a**(6) | Nausea (1-7 Likert scale (1=great improvement, 7=severe deterioration)) | I: 2.5±1.27 // C:3.75±1.16 | Difference in the outcome between both groups at the end of study period:  -p=0.003 |
| **Nausea** | | | |
| **Naftali 2021a**(6) | Nausea (1-7 Likert scale (1=great improvement, 7=severe deterioration)) | I: 3.25±1.58 // C:3.9±1.02 | Difference in the outcome between both groups at the end of study period:  -p=0.76 |
| **Appetite** | | | |
| **Naftali 2021a**(6) | Appetite (1-7 Likert scale (1=great improvement, 7=severe deterioration)) | I: 2.55±1.31 // C:3.6±1.09 | Difference in the outcome between both groups at the end of study period:  -p=0.013 |

# References

1.	The Health Effects of Cannabis and Cannabinoids: The Current State of Evidence and Recommendations for Research [Internet]. Washington, D.C.: National Academies Press; 2017 [cited 2024 Dec 18]. Available from: https://www.nap.edu/catalog/24625

2.	Norton C, Czuber-Dochan W, Artom M, Sweeney L, Hart A. Systematic review: interventions for abdominal pain management in inflammatory bowel disease. Aliment Pharmacol Ther. 2017 July;46(2):115–25.

3.	Kafil TS, Nguyen TM, MacDonald JK, Chande N. Cannabis for the treatment of Crohn’s disease. Cochrane Database Syst Rev. 2018 Nov 8;11(11):CD012853.

4.	Institute of Medicine (US). Marijuana and Medicine: Assessing the Science Base [Internet]. Joy JE, Watson SJ, Benson JA, editors. Washington (DC): National Academies Press (US); 1999 [cited 2024 June 26]. Available from: http://www.ncbi.nlm.nih.gov/books/NBK230716/

5.	Naftali T, Bar-Lev Schleider L, Dotan I, Lansky EP, Sklerovsky Benjaminov F, Konikoff FM. Cannabis induces a clinical response in patients with Crohn’s disease: a prospective placebo-controlled study. Clin Gastroenterol Hepatol Off Clin Pract J Am Gastroenterol Assoc. 2013 Oct;11(10):1276-1280.e1.

6.	Naftali T, Bar-Lev Schleider L, Almog S, Meiri D, Konikoff FM. Oral CBD-rich Cannabis Induces Clinical but Not Endoscopic Response in Patients with Crohn’s Disease, a Randomised Controlled Trial. J Crohns Colitis. 2021 Nov 8;15(11):1799–806.

7.	Naftali T, Schleider LBL, Benjaminov FS, Konikoff FM, Matalon ST, Ringel Y. Cannabis is associated with clinical but not endoscopic remission in ulcerative colitis: A randomized controlled trial. PLOS ONE. 2021 Feb 11;16(2):e0246871.

8.	Irving PM, Iqbal T, Nwokolo C, Subramanian S, Bloom S, Prasad N, et al. A Randomized, Double-blind, Placebo-controlled, Parallel-group, Pilot Study of Cannabidiol-rich Botanical Extract in the Symptomatic Treatment of Ulcerative Colitis. Inflamm Bowel Dis. 2018 Mar 19;24(4):714–24.
